# Percutaneous biliary drainage: a superior option in select cases of acute cholangitis: a case report

**DOI:** 10.1093/jscr/rjaf178

**Published:** 2025-04-03

**Authors:** Yavor Assenov, Ivan Vasilev, Boris Kunev

**Affiliations:** Department of Surgery, University Hospital “Tsaritsa Joanna – ISUL”, Medical University of Sofia, “Byalo more 8” Str., Sofia 1527, Sofia, Bulgaria; Department of Surgery, University Hospital “Tsaritsa Joanna – ISUL”, Medical University of Sofia, “Byalo more 8” Str., Sofia 1527, Sofia, Bulgaria; Department of Surgery, University Hospital “Tsaritsa Joanna – ISUL”, Medical University of Sofia, “Byalo more 8” Str., Sofia 1527, Sofia, Bulgaria

**Keywords:** acute cholangitis, percutaneous biliary drainage, ERCP, biliary obstruction, multidisciplinary approach

## Abstract

Acute cholangitis is a severe, potentially life-threatening condition, frequently occurring post-ERCP. While endoscopic drainage is the preferred first-line treatment, percutaneous biliary drainage (PTBD) is a crucial alternative in select cases. We present a 55-year-old patient with prior left hemicolectomy and liver metastasis treatment developed acute cholangitis following failed ERCP stent placement, leading to rapid deterioration. Due to high anesthetic risk, ultrasound-guided PTBD with an 8 Fr pigtail catheter was performed under local anesthesia, resulting in significant clinical improvement. Broad-spectrum antibiotics targeted *Escherichia coli*. The misplaced stent was subsequently replaced, and the patient was discharged on postoperative Day 9 with normalized bilirubin and coagulation. Follow-up confirmed good drain tolerance and recovery. This case underscores PTBD’s critical role when endoscopic drainage fails. A multidisciplinary approach and early intervention are essential to improving outcomes in acute cholangitis management.

## Introduction

Acute cholangitis is a significant and potentially life-threatening complication following ERCP, affecting ~1%–5% of cases [1, 2]. Clinical manifestations, such as fever, jaundice, and pain (Charcot triad), along with hypotension and confusion (Reynolds pentad), serve as well-established diagnostic indicators for acute cholangitis [1]. The incidence of this syndrome varies based on the nature of the stenosis, with hilar stenosis being particularly severe, affecting ~21% of cases [3]. Nearly half of patients with hilar stenosis develop moderate to severe cholangitis, necessitating additional biliary drainage interventions. Historical data reveals mortality rates ranging from 2% to an alarming 65%, highlighting the urgency of prompt diagnosis and precise therapeutic intervention [1, 4]. While current guidelines advocate for endoscopic drainage as the primary approach, percutaneous drainage emerges as a preferred alternative in carefully selected cases. In this report, we present a case where a percutaneous approach was deemed the optimal course of action.

## Case report

We present the case of a 55-year-old patient who underwent a left hemicolectomy in 2021 due to adenocarcinoma. Subsequent procedures included an atypical resection addressing liver metastasis in segment 6 and microwave ablation in segment 8 in 2022. Despite ongoing chemotherapy, the patient developed jaundice, prompting referral to the gastroenterological department.

A CT scan revealed a sizable tumor mass in the hepatoduodenal ligament involving the hepatic confluence, with additional metastases in segments 4 and 8. Radiofrequency ablation of the lesion in segment 8 was performed. An ERCP was performed, and a self-expandable metal stent was placed. However, the patient's condition rapidly deteriorated post-procedure, marked by fever (>38°C), aggravated jaundice, elevated ASAT, ALAT levels, and an INR exceeding 3. Subsequent endoscopic intervention revealed misplacement of the stent into the right hepatic duct, failing to drain the left lobe. Due to the patient's unstable condition, anesthesia was deemed high-risk and repeated endoscopic attempts were unsuccessful.

**Figure 1:**
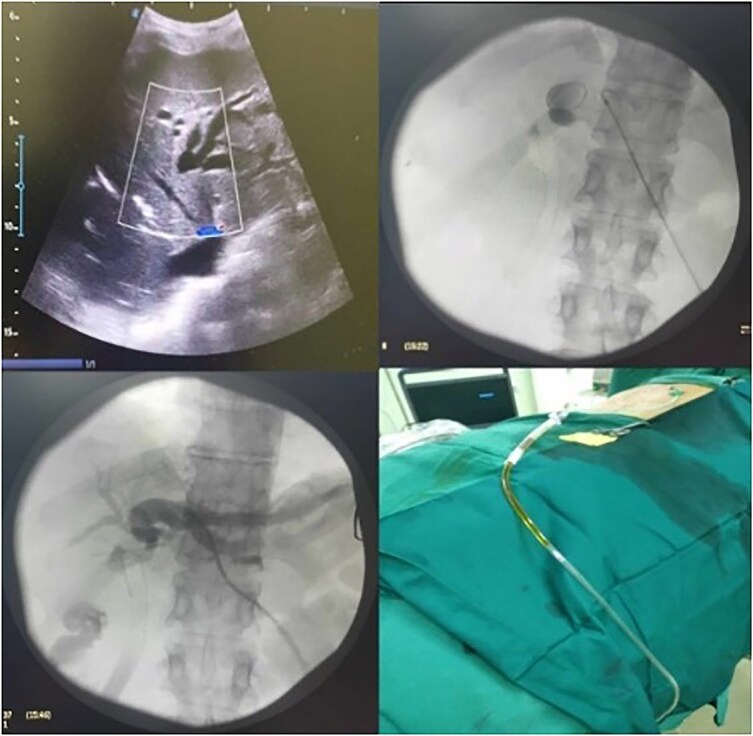
Ultrasound exploration and PTBD placement.

Ultrasound confirmed extensive biliary tree dilatation in the left liver lobe, prompting a decision for percutaneous trans-hepatic biliary drainage (PTBD) under ultrasound guidance and local anesthesia. After successful cannulation, an 8 Fr pigtail catheter was placed, accompanied by aggressive fluid resuscitation and broad-spectrum antibiotics. Microbiological analysis identified *Escherichia coli* infection, sensitive to the administered antibiotics, leading to continuation of the antibiotic regimen for 7 days (Fig. 1).

Following PTBD, the patient's condition significantly improved, leading to discharge on the 9th postoperative day. After 14 days, the patient demonstrated good tolerance to the PTBD drain, with normalized bilirubin levels and coagulation status. Subsequent admission to a gastroenterological department at another hospital involved the removal of the misplaced stent and the placement of new stents in both the left and right hepatic ducts (double barrel).

## Discussion

Acute cholangitis is recognized as the most prevalent infectious complication following ERCP-related procedures, with its occurrence notably heightened by stent placement and malignant stenosis. The literature documents a broad spectrum of incidence rates, ranging from 3.5% to 40% with stent placement and ~15.6% in cases of malignant obstruction. Recent studies suggest an overall incidence of endoscopically-related cholangitis at 21.5%, with more than half of patients experiencing moderate to severe manifestations [3]. Furthermore, individuals affected by cholangitis often demonstrate diminished rates of clinical success, elevated early mortality, shorter stent patency, and reduced overall survival when compared to counterparts without cholangitis [1, 3].

There is an association between drainable liver volume and stenting success, with cholangitis being more prevalent when a drained liver volume exceeds 50% [1, 3]. Management of acute cholangitis resulting from occlusion by self-expandable metallic stents in patients with malignant biliary obstructions demands prompt attention due to the potential rapid progression to severe stages and accompanying organ dysfunction. Hence, early detection is crucial for initiating emergency biliary drainage and medical therapy. For diagnostic and severity staging, the 2018 Tokyo Guidelines criteria are utilized. These guidelines offer a standardized approach to assessing the severity of acute cholangitis, facilitating accurate diagnosis and risk stratification [5].

Historical studies from the 1960s revealed alarming mortality rates exceeding 50% for severe cholangitis. Nevertheless, advancements in diagnostic techniques and improvements in medical care have contributed to a significant reduction in mortality rates. Reports from the 1990s observed rates ranging between 11% and 27%, while more recent studies indicate even lower mortality rates of 10% or less [1, 6, 7].

Several mechanisms counteract potential infections in the biliary system. The Sphincter of Oddi, for instance, mechanically protects against ascending infections, while bile itself acts as a bactericidal agent by washing bacteria into the duodenum. Additional protective mechanisms include the production of IgA and mucus by the endothelium, as well as the presence of tight junctions between cells and Kupffer cells that directly inhibit bacterial translocation. Furthermore, the salt content in bile suppresses bacterial reproduction. However, disruptions in these protective mechanisms can lead to infection [1, 8].

In our presented case, biliary stenosis resulted in stasis and increased intraductal pressure. The unsuccessful attempt at endoscopic stenting introduced infection, further complicating matters as the infected bile was blocked in the undrained segments of the left liver lobe (segments 2, 3, and 4), leading to systemic inflammatory response syndrome.

In cases of severe cholangitis due to isolated segmental blockage from failed endoscopic manipulation, the percutaneous approach is a viable option. A significant advantage is that percutaneous drainage can be performed under local anesthesia, particularly beneficial for unstable patients [9]. Prompt drainage is paramount in patients with severe acute cholangitis to save lives, with a delay in manipulation beyond 48 hours associated with poor prognosis [10]. Additionally, adequate drainage can reduce the duration of necessary antibiotic therapy [1].

## Conclusion

Severe acute cholangitis poses a significant clinical challenge, demanding rapid diagnosis and timely intervention. In instances where endoscopic drainage proves unsuccessful or unfeasible, percutaneous drainage emerges as a crucial alternative. This case underscores the importance of a multidisciplinary approach, prompt recognition of complications, and the critical role of percutaneous interventions in managing acute cholangitis. Further research and clinical experience are warranted to refine treatment algorithms and improve outcomes in this complex clinical scenario.

## Data Availability

The data sets supporting the conclusions of this article are included in the article.
